# Development and fecundity performance of *Grapholita molesta* and *Grapholita dimorpha* (Lepidoptera: Tortricidae) on different immature fruits

**DOI:** 10.1371/journal.pone.0217492

**Published:** 2019-05-31

**Authors:** Souvic Sarker, Un Taek Lim

**Affiliations:** Department of Plant Medicals, Andong National University, Andong, Republic of Korea; Northwest A&F University, CHINA

## Abstract

Both *Grapholita molesta* (Busck) and *Grapholita dimorpha* Komai (Lepidoptera: Tortricidae) are important pests of pome and stone fruits. Mature fruits of peach and plum have been tested as food sources for *G*. *molesta*, but no studies have examined the suitability of immature fruits, which are the fruit stage more likely to be available for the spring populations of both *G*. *molesta* and *G*. *dimorpha*. Thus, we evaluated immature fruits of peach, plum, and apple as food sources by assessing their effects on biological and behavioral attributes of these moths in the laboratory. Immature fruits were collected in May and June of 2016 and again in 2017. The first-instar larvae of *G*. *molesta* preferred either peach or plum, which showed exit rates of mature larvae of 81.0 and 100.0% for the two fruits, respectively. On peach, development time was shorter, and fecundity was lower than plum. However, *G*. *dimorpha* preferred plum and apple, showing the lowest fruit-boring rate and lowest mature larval exit rate on peach, from which only one female moth emerged but produced no eggs. In conclusion, it seems that at the immature fruit stage, plum and apple are better food sources for both *G*. *molesta* and *G*. *dimorpha* than is peach.

## Introduction

Among the microlepidopterans, the Tortricoidea is one of the most diverse superfamilies [1]. Tortricoidea contains a single family (Tortricidae), comprising over 9,800 species in 1,050 genera (http://www.tortricidae.com). *Grapholita molesta* (Busck) and *Grapholita dimorpha* Komai belong to the subfamily of Olethreutinae and are among the most destructive of fruit pests [1–8]. *Grapholita molesta* (Oriental fruit moth) is an invasive oligophagous insect of Asian origin that reproduces on Rosaceous plants, having 3–6 generations per year [8]. The main hosts of Oriental fruit moth are species of *Prunus* (*Rosaceace*, subfamily *Drupaceae*) including peaches, nectarines, plums, and apricots [9]. This moth is widely distributed throughout the temperate and subtropical regions of the world [8, 10, 11, 12, 13, 14]. Another closely related species, *G*. *dimorpha* (the plum fruit moth) was first reported by Komai [3] and is now found in many northeast Asia regions, including Japan, Korea, China, and Russia [2, 7, 15, 16]. *Grapholita dimorpha* is reported as a pest of plum, pear, and apple [17–19]. The female *G*. *dimorpha* lays one or two eggs on the fruit surface, and the larva makes a pinhole in the fruit skin after hatching and then bores into the fruit [5]. As the pinhole is small, it is very difficult to distinguish damaged from undamaged fruits at harvest [18].

Immature fruits in spring may not support complete larval development, and larvae may need to move to other food sources [20]. For lepidopteran larvae, olfaction can be an important cue for orientation to the feeding substrate [21]. Lepidopteran larvae have shown concentration-dependent and species-specific behavioral responses to different feeding substrates or odors [21, 22]. However, no information is available on the host exploitation behavior of *G*. *molesta* and *G*. *dimorpha* on immature host fruits as spring food sources. We evaluated host preference of *G*. *molesta* and *G*. *dimorpha* larvae on immature fruits. In many parts of its geographic range, *G*. *molesta* shows a shift from peach orchards in spring to apple or pear orchards in later season due to earlier maturation of stone fruits [8, 14, 23, 24, 25]. Many studies on larval survival and phenological patterns have been conducted using mature fruits [8], but diet suitability of immature fruits is barely known. Thus, we conducted life table studies using immature fruits, including peach, plum, and apple, as diets during the spring period, comparing development times, survivorship, and reproduction of both *G*. *molesta* and *G*. *dimorpha* on various immature fruits.

## Materials and methods

### Insect and rearing procedures

Apples infested with oriental fruit moth (*G*. *molesta*) and plum fruit moth (*G*. *dimorpha*) were collected at Andong in 2016 and kept in ventilated plastic containers (24.0 L × 17.0 W × 8.0 H cm) at 24.9 ± 0.1°C and 50.2 ± 1.3% RH in an incubator (DS-11BPL, Dasol Scientific Co., Ltd, Hwaseong, Gyeonggi, Republic of Korea). Mature 5^th^ instar larvae emerged from the apples and spun cocoons in paper toweling provided for pupation. Pupae were collected as they became available and put inside a breeding dish (10.0 D × 4.0 H cm, 310102, SPL, Pocheon, Republic of Korea). When adults emerged, they were transferred into a ventilated acrylic cage (25.5 H × 8.5 D cm) and a piece of cotton soaked with 10% sugar solution was provided as a food source in each cage. These cylindrical cages were kept in a desiccator (36.0 L × 28.0 W × 25.0 H cm) at 25.6 ± 0.1°C and 91.2 ± 0.1% RH (16:8 L:D) in the incubator. When the moths started to lay eggs on the walls of the cylinders, cages were changed daily to collect age-specific cohorts of eggs. Acrylic cylinders with eggs on the walls were held in a separate incubator until egg hatch, and the first-instar larvae were collected for life table experiments or further mass rearing. We reared them five generations while doing the experiments with intermittent addition of wild male population to reduce inbreeding depression.

### Plant materials

Three types of immature fruits (peach, plum, and apple) were used in this study. Fruits of peach [*Prunus persica* (L.) “Local variety (unknown)”], plum (*Prunus domestica* L.variety “Royal Daeseok), and apple [*Malus domestica* Borkh. Variety “Fuji” (strain ‘Busa’)] were collected from Dosan, Iljeek, and Gilan County, respectively, Andong City, Republic of Korea in both 2016 and 2017. Peach fruits were collected on 25 May, plum fruits collected on 12 and 25 May, and apple fruits on 14 and 6 June in 2016 and 2017, respectively. All orchards were free from pesticide application. The diameter of all fruits used in the life table study were measured and averaged 1.4 ± 0.03 (peach), 2.4 ± 0.1 (plum), and 2.4 ± 0.02 (apple) cm. Similarly, fruits used in the choice test averaged 1.4 ± 0.02 (peach), 2.3 ± 0.1 (plum), and 2.3 ± 0.01 (apple) cm. After fruits were collected from orchards, they were sealed in plastic zipper bags, and kept under 4°C in a refrigerator and used within two days.

### Development and survival rate of immature stages

Field-collected fruits used in this experiment were placed in a breeding dish (10.0 D × 4.0 H, 310102, SPL, Pocheon, Republic of Korea) and one larva (<6h old) of *G*. *molesta* or *G*. *dimorpha* was released on each fruit at 25.1 ± 0.1°C and 42.2 ± 0.3% RH in the incubator. In total, there were 76 larvae for peach, 52 for plum, and 36 for apple for *G*. *molesta* and 30 for peach, 32 for plum, and 30 for apple for *G*. *dimorpha* in the assay. When a mature larva emerged from its fruit, it was transferred into another breeding dish filled with tissue paper for pupation. Duration of the larval stage was measured as the time from larval emergence from the egg (start of experiment) to emergence of the mature larva from fruits; duration of the prepupal stage was defined as the period from emergence of the mature larva from the fruit to pupation; and the duration of the pupal stage was from pupation to adult emergence.

### Longevity and fecundity of adult female

Newly emerged adults were transferred to transparent square breeding dishes (7.2 L × 7.2 W × 10.0 H cm SPL, Pocheon, Republic of Korea, each with three 40 mm-mesh screens; one per side), with one male and one female moth per dish. A cotton wick soaked in 10% sugar solution was placed in each container to provide a carbohydrate source for adult feeding. Newly laid eggs on the surface of the container were marked and recorded daily until the death of adult female. Square breeding dishes were replaced with new ones daily to avoid pathogenic infection. At the end of the experiment, unmated females were identified by the absence of a spermatophore in their eggs and were excluded from the analysis [8] and a total of 17 replications for peach, 18 for plum, and 9 for apple against *G*. *molesta* and 1 replications for peach, 8 for plum, and 10 replications against apple for *G*. *dimorpha* were used in this study.

### Choice test of *G*. *molesta* and *G*. *dimorpha*

Peach, plum, and apple fruits collected as described above were used as food sources for this experiment. To determine larval preference among the three fruits (peach, plum, and apple), one fruit of each type was placed in a triangular pattern in a plastic tray (25.0 D cm) at a distance of10 cm from the center. The distance between fruits was 17 cm. One newly emerged first instar larva (<5h old) of either *G*. *molesta* or *G*. *dimorpha* was placed in the center of the triangle, and then food-exploitation larval behaviors were observed visually. Observations were made for 1h at 28.6 ± 0.5°C and 47.1 ± 6.5% RH in the laboratory. The final choice made by each tested larva was determined to be whatever fruit the larva selected if it stayed on the fruit for more than 1 hour and giving up rate was recorded if the larva left the fruit before 1 hour.

### Statistical analysis

Differences in developmental time, the length of the preoviposition period, fecundity, and longevity of *G*. *molesta* and *G*. *dimorpha* reared on different fruits were analyzed with one-way ANOVA. From peach, only one female adult of *G*. *dimorpha* emerged and it died without laying any eggs, thus we discarded the data of fecundity on peach from the analysis. The egg hatch rate, the first instar larval boring rate, the mature larval exit rate, the pupation rate, emergence rate, larval mortality, and adult mortality were analyzed using Chi-square tests with a post-hoc multiple comparison test analogous to Tukey’s test [26].

The jackknife procedure was conducted to test the differences in population parameters, i.e., net production rate (*R*_*O*)_, mean generation time (*T*), intrinsic rate of increase (*r*_*m*_), doubling time (*Dt*), and finite rate of increase (*λ*) [27]. The survival rate (*Sxj*) (*x* = age, *j* = stage) is the probability that a newly laid egg would survive to age *x* and stage *j*. Algorithms for jackknife estimation of the means and variances, and the construction of confidence intervals are described only for *Ro* (The net contribution from each female to the next generation, which is expressed as the total of female offspring per female during the entire oviposition period) [27]. The same procedures were used for the other parameters (*r*_*m*_, *T*, *Dt*, and *λ*). All data related to fertility in the life tables were entered into a computer program (LIFETABLE.SAS) [27] and analyzed using SAS 9.3 (SAS Institute 2010).

Choice rates among fruits were compared by Chi-square tests and a post-hoc multiple comparison test analogous to Tukey’s test use to separate means. Time taken to choose a food source was analyzed with one-way ANOVA.

## Results

### Effect of fruit type on immature developmental rates of *Grapholita molesta* and *G*. *dimorpha*

**Fig 1:**
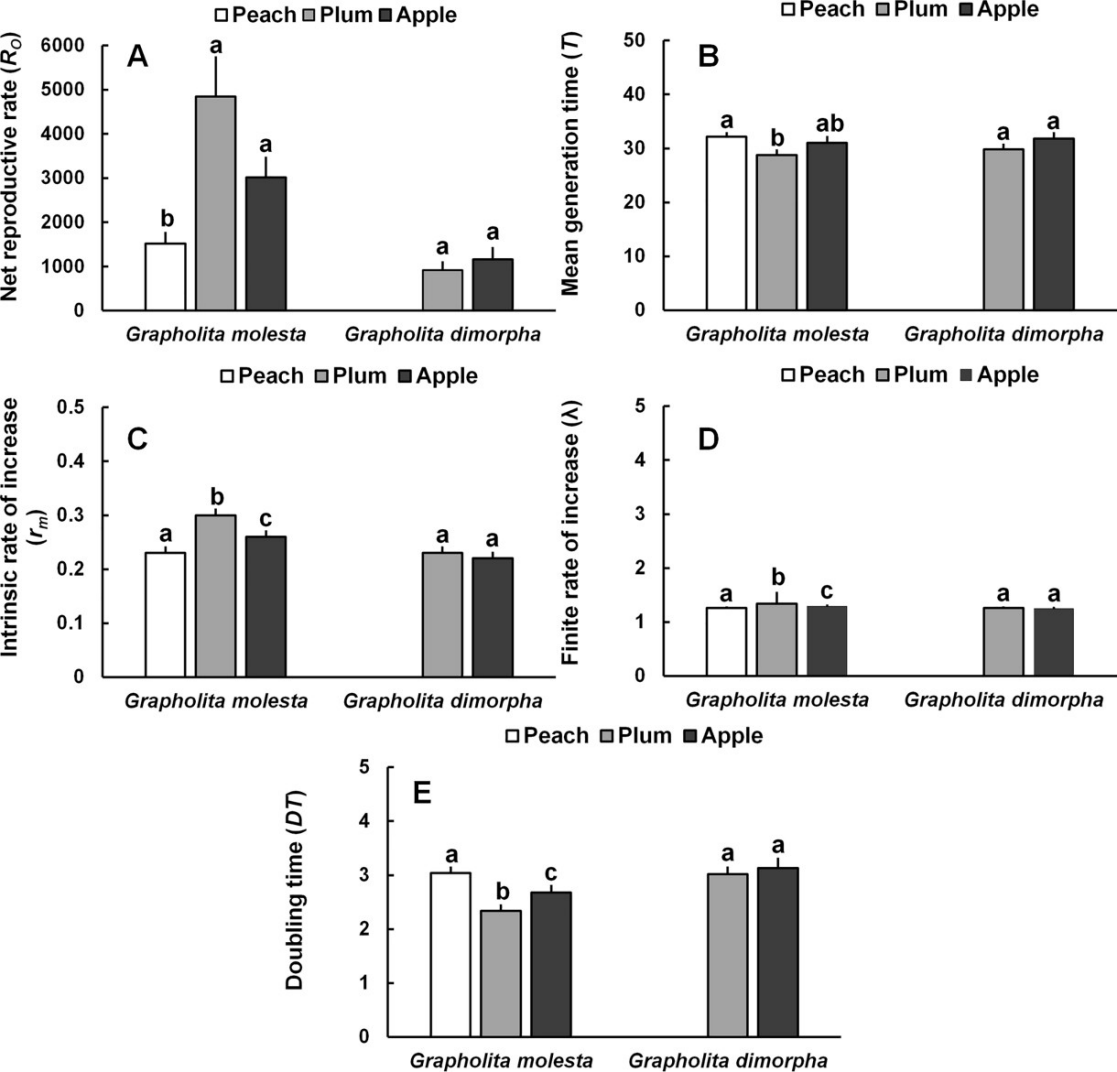
Duration (d ± SE) of each stage of *Grapholita molesta* (n = 76 for peach, n = 52 for plum, and n = 36 for apple) and *Grapholita dimorpha* (n = 30 for peach, n = 32 for plum, and n = 30 for apple) reared on different fruits under laboratory conditions. A) Egg stage, B) Larval stage, C) Prepupal stage, D) Pupal stage, and E) Immature stage.

Significant differences in the duration of each life stage were only found in the larval stage, with larvae developing more rapidly on peach and plum than on apple (*F* = 8.726; *df* = 2, 123; *P <* 0.001) (Fig 1). No significant differences among fruits were found for developmental times for any other life stages, i.e., egg (*F =* 0.214; *df* = 2, 20; *P* = 0.815), prepupa (*F =* 0.723; *df* = 2, 113; *P* = 0.487), pupa (*F* = 1.414; *df* = 2, 101; *P* = 0.247), and immature stage in total (*F* = 0.978; *df* = 2, 101; *P* = 0.379).

For *G*. *dimorpha*, there were no significant differences in duration of the egg stage (*F =* 0.350; *df* = 2, 18; *P* = 0.707), the prepupal stage (*F =* 0.760; *df* = 2, 45; *P* = 0.475), or the pupal stage (*F =* 0.240; *df* = 2, 38; *P* = 0.709) (Fig 1). However, there was a significant difference in the larval developmental time, with larvae developing most rapidly in plum (*F =* 12.810; *df* = 2, 49; *P* < 0.001), corresponding to the diet preference shown by the 1^st^ instar larva (Fig 1).

**Fig 2:**
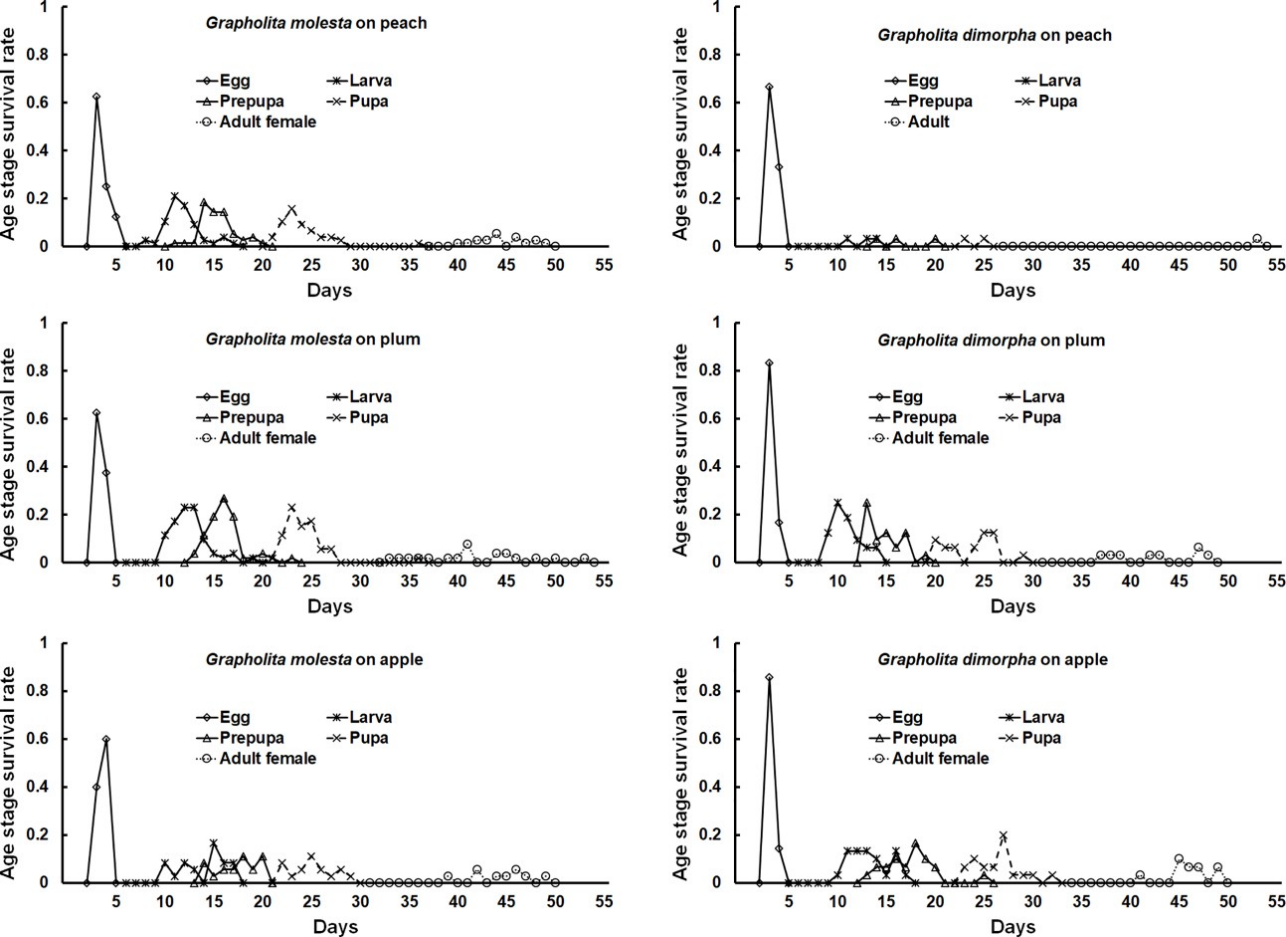
Larval and pupal mortality of *Grapholita molesta* and *Grapholita dimorpha*.

For *G*. *molesta*, larval mortality of *G*. *molesta* was lowest on plum (5.8%) compared with peach (28.9%) or apple (41.7%) (*χ*^*2*^ = 16.453; *df* = 2; *P <* 0.001; Fig 2). In addition, their developmental times were also shorter on plum than apple. The boring rate of neonatal larvae was significantly higher on plum (94.0%) and peach fruits (86.0%) compared with apple (64.0%) (*χ*^*2*^ = 15.452; *df* = 2; *P <* 0.001; Table 1). The exit rate from fruit of mature larvae was also higher on plum (100.0%) than on peach (81.0%) (*χ*^*2*^ = 10.268; *df* = 2; *P* = 0.006). However, the egg hatch rate was higher on plum (90.0%) and apple (94.0%) than peach (85.0%) (*χ*^*2*^ = 10.898; *df* = 2; *P* = 0.004). No significant difference was found among fruits for either the pupation rate (*χ*^*2*^ = 2.249; *df* = 2; *P =* 0.325) or adult emergence rate (*χ*^*2*^ = 0.013; *df* = 2; *P =* 0.994).

**Table 1:** Survival rate of each stage of *Grapholita molesta* and *Grapholita dimorpha* provided with different fruits under laboratory conditions.

Insect	Fruit type	Hatching rate	Boring rate	Exiting rate	Pupation rate	Emergence rate
*G*. *molesta*	Peach	0.85 (266/312) b	0.86 (66/76) a	0.81 (54/66) b	0.90 (49/54) a	0.90 (44/49) a
	Plum	0.90 (546/603) a	0.94 (49/52) a	1.00 (49/49) a	0.95 (47/49) a	0.89 (42/47) a
	Apple	0.94 (207/221) a	0.64 (23/36) b	0.91 (21/23) ab	0.86 (18/21) a	0.89 (16/18) a
*G*. *dimorpha*	Peach	-	0.20 (6/30) b	0.50 (3/6) b	1.00 (3/3) a	0.67 (2/3) a
	Plum	91.48(204/223) a	0.78 (25/32) a	1.00 (25/25) a	0.88 (22/25) a	0.82 (18/22) a
	Apple	90.87(189/208) a	0.77 (23/30) a	0.96 (22/23) a	0.95 (21/22) a	0.90 (19/21) a

Means within a column with different letters are significantly different (*P* < 0.05)

Interestingly, for *G*. *dimorpha*, larval mortality was also lowest on plum (16.7%) followed by apple (26.6%) and peach (45.0%) (*χ*^*2*^ = 38.430; *df* = 2; *P<* 0.001; Fig 2). No significant difference in pupal mortality was found among fruits (*χ*^*2*^ = 1.441; *df* = 2; *P =* 0.487). In contrast to results for *G*. *molesta*, the boring rate of the 1^st^ instar *G*. *dimorpha* larva on peach (20.0%) was lower than plum (78.0%) or apple (77.0%) (*χ*^*2*^ = 27.508; *df* = 2; *P <* 0.001; Table 1). The exit rate of mature larvae from fruits was also lowest in peach (50.0%) (*χ*^*2*^ = 18.184; *df* = 2; *P <* 0.001). However, there was no significant difference among fruits in pupation rate (*χ*^*2*^ = 1.161; *df* = 2; *P =* 0.559) or emergence of mature larvae from fruits (*χ*^*2*^ = 1.441; *df* = 2; *P =* 0.487).

### Evaluation of different fruits on adult longevity or female fecundity

**Fig 3:**
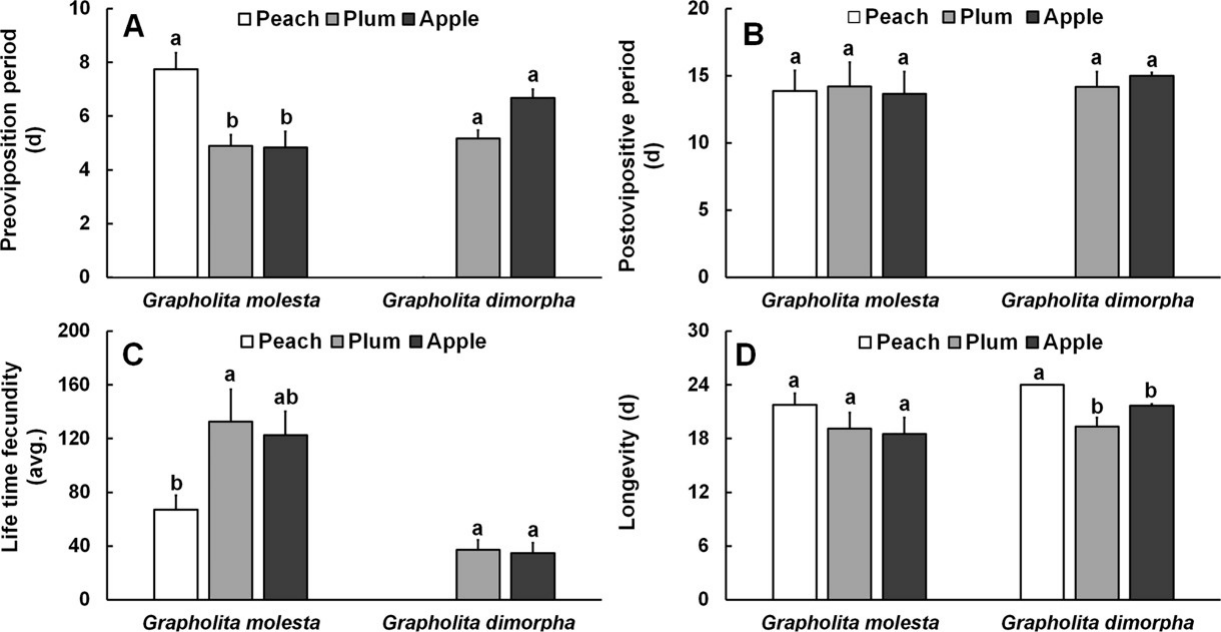
Fecundity (Mean ± SE) of female adult of *Grapholita molesta* and *Grapholita dimorpha* reared on different fruits under laboratory conditions. A) Preoviposition period, B) Postoviposition period, C) Lifetime fecundity, and D) Longevity.

For *G*. *molesta*, diet significantly affected the duration of the preoviposition period (*F* = 8.276; *df* = 2, 22; *P* = 0.002) and lifetime fecundity (*F* = 3.457; *df* = 2, 22; *P* = 0.051) (Fig 3). The duration of the preoviposition period was greatest on peach compared with plum and apple. Fecundity of females reared on plum or apple was higher than that of those reared on peach. No significant differences were observed in post-oviposition survival period (*F* = 0.034; *df* = 2, 22; *P* = 0.973) or total adult longevity (*F* = 1.031; *df* = 2, 22; *P* = 0.377).

**Fig 4:**
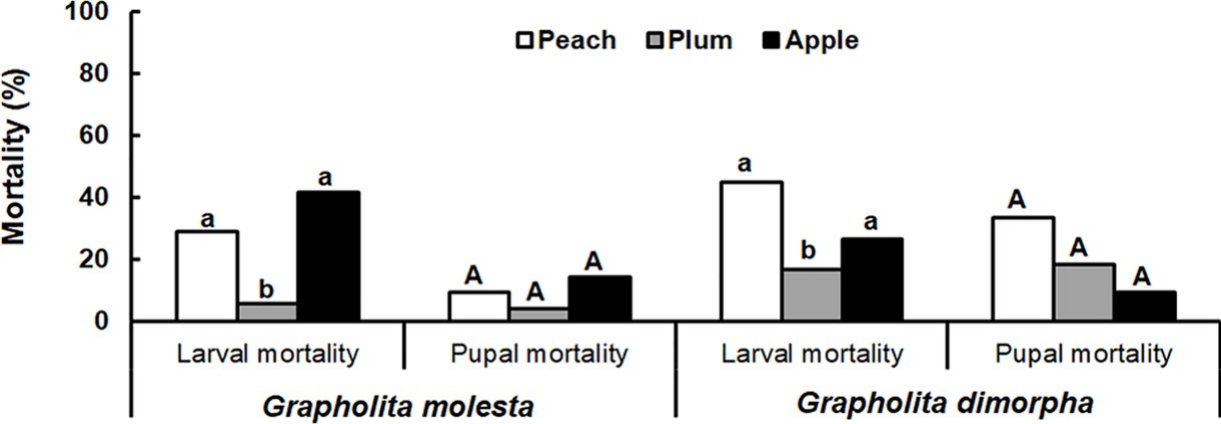
Age-stage survival (*Sxj*) of *Grapholita molesta* and *Grapholita dimorpha* on peach, plum, and apple fruit.

For *G*. *dimorpha*, females reared on peach never laid any eggs (Fig 3). There was no significant difference in the length of the preoviposition period between plum and apple (*F* = 31.670; *df* = 2, 12; *P* <0.001). The duration of the post-oviposition period was also not different between plum and apple (*F* = 24.290; *df* = 2, 12; *P* <0.001). Diet caused no significant difference in fecundity (*F* = 0.060; *df* = 1, 11; *P* = 0.817), but a significant difference was found among fruits for longevity (*F* = 10.450; *df* = 2, 12; *P* = 0.004). Longevity of females developing on peach (24.0 d) was significantly greater than that of moths reared on plum or apple. Age- and stage- specific survival rates of *G*. *dimorpha* were lowest on peach in the larval, pupal, and adult stages (Fig 4).

### Life history of *G*. *molesta* and *G*. *dimorpha*

**Fig 5:**
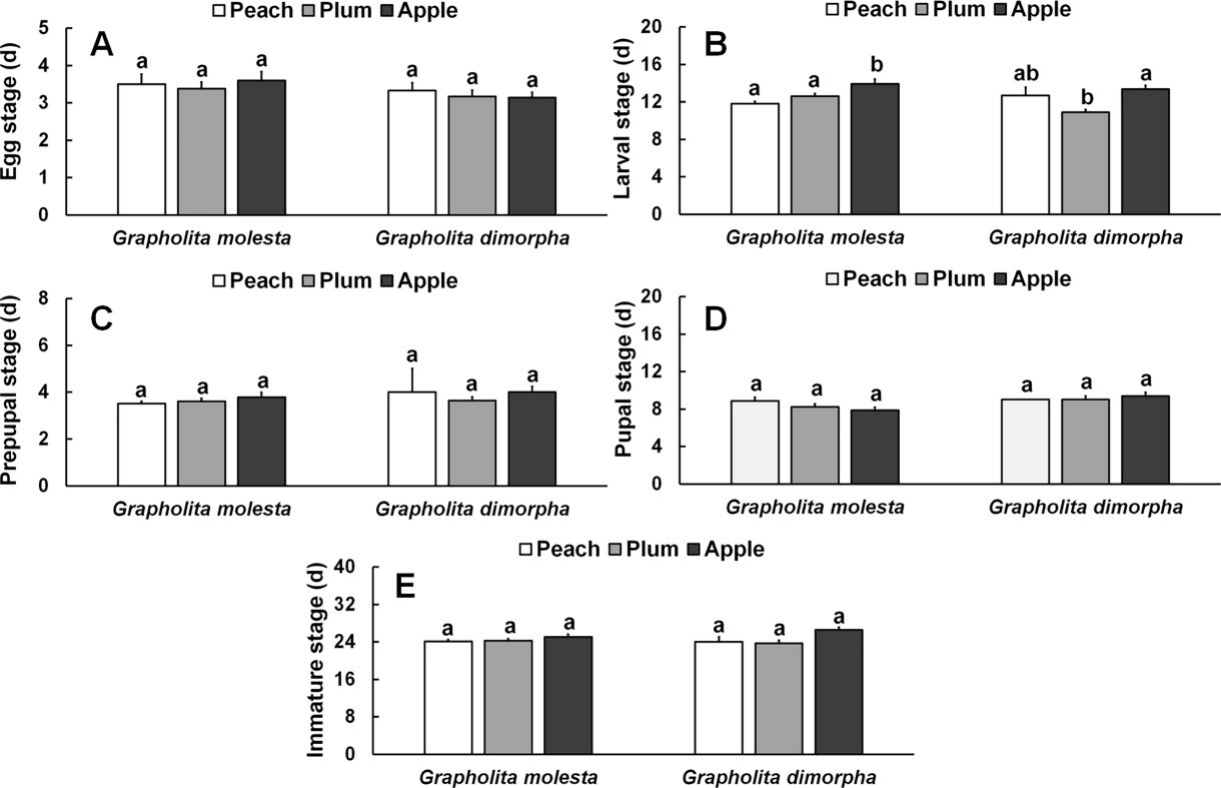
Life table parameters of *Grapholita molesta* and *Grapholita dimorpha* reared on different fruits. A) Net productive rate, B) Mean generation time, C) Intrinsic rate of increase, D) Finite rate of increase, and E) Doubling time. Means followed by the same letter in a column are not significantly different, Student’s *t*-test for pairwise group comparison at P<0.05. *R_O_*, Net reproductive rate; T, Mean generation time; DT, Doubling time; λ, Finite rate of increase; *r_m_*, Intrinsic rate of increase.

For *G*. *molesta*, significant differences were observed among fruits in net reproductive rate (*R*_*O*_), mean generation time (*T*), intrinsic rate of increase (*r*_*m*_), finite rate of increase (*λ*), and doubling time (*DT*). The *R*_*O*_ value was significantly lower for moths when reared on peach, while the *T* value was significantly longer (i.e., slower developmental rate) in peach than other fruits. The *r*_*m*_ value was significantly lower in peach and the *DT* value also significantly higher (i.e., slower population growth) in peach than in moths reared on plum or apple (Fig 5).

For *G*. *dimorpha*, which did not lay eggs after being reared on peach, there was no significant difference in any of the life table parameters between plum and apple (Fig 5).

### Choice test

Larvae of *G*. *molesta* showed no specific preference among fruits in terms of first larval choice (*χ*^*2*^ = 4.262; *df* = 2; *P* = 0.119). Also, there was no statistically significant difference in the time taken to encounter the first choice fruit (*F* = 1.914; *df* = 2, 30; *P =* 0.172) (Table 2). However, 38% of the larvae gave up plum fruit as their first choice (*χ*^*2*^ *=* 9.536; *df* = 2; *P* = 0.009), and, overall, peach was the most preferred fruit (*χ*^*2*^ *=* 6.931; *df* = 2; *P =* 0.031). For *G*. *dimorpha*, the first- instar larvae preferred plum fruit as their first choice (*χ*^*2*^ *=* 13.418; *df* = 2; *P* = 0.001), while they gave up peach fruits at 33.0% as their first choice before the one hour mark (used as index of fruit acceptance). Overall, *G*. *dimorpha* preferred plum fruits over apple or peach (*χ*^*2*^ *=* 18.600; *df* = 2; *P <*0.001) (Table 2).

**Table 2:** Feeding behavior of *Grapholita molesta* (n = 40) and *Grapholita dimorpha* (n = 33) on three fruits during a one hour choice test.

Insect	Fruit type	First choice rate	Time to reach first choice(min)	Giving-up rate of the first choice	Second choice rate	Time to reach second choice(min)	Final choice rate
*G*. *molesta*	Peach	0.38 (15/40) a	20.07 ± 3.59 a	0.00 (0/15) a	0.00 a	0.00	0.51 (15/29) a
	Plum	0.20 (8/40) a	24.00 ± 3.83 a	0.38 (3/8) bc	0.03(1/31) a	6.00	0.21 (6/29) b
	Apple	0.20 (8/40) a	12.75 ± 2.23 a	0.00 (0/8) ac	0.00 a	0.00	0.28 (8/29) ab
*G*. *dimorpha*	Peach	0.09 (3/33) b	34.67 ± 11.32 a	0.33 (1/3)	0.0	3.00	0.10 (2/20) b
	Plum	0.42 (14/33) a	30.42 ± 4.54 a	0.0	0.0	0.00	0.70 (14/20) a
	Apple	0.12 (4/33) b	19.50 ± 8.57 a	0.0	0.0	0.00	0.20 (4/20) b

Means within a column with different letters are significantly different (*P* < 0.05)

## Discussion

Host plant availability is a major factor determining the population dynamics of herbivorous insects [8, 28, 29]. Larval development rate, development time, survival rate, and fecundity are affected by the physical characteristics as well as chemical components of the host plants [25, 28, 30, 31, 32, 33, 34]. Population dynamics of phytophagous insects is significantly influenced by the diversity of plant species and the nutritional levels of plant tissues [35]. The ability of feeding on a wide variety of hosts is a common feature among various invasive herbivores [14, 36, 37].

Although Myers et al. [32] suggested that larval survival of *G*. *molesta* is better on ripening peach fruit, the effect of an immature fruit diet on larval development and reproduction has never been determined, and few studies exist on the basic biology, ecology, or dietary preferences of *G*. *dimorpha*. The present study shows that the type of fruit significantly affects the larval development, reproduction, and life table parameters of both *G*. *molesta* and *G*. *dimorpha*. In the choice test in this study, first-instar larvae of *G*. *molesta* most frequently accepted peach as their final choice, whereas *G*. *dimorpha* chose plum. Wills et al. [38] found that sucrose content is generally higher in peach (3.1–5.5 g 100 g-l) than plum fruits (1.0–3.4 g 100 g-l), thus sugar content alone may not be the factor responsible for this asymmetrical choice between fruits by our two study species. Semiochemicals emitted from the fruit might also have affected their choice [39]. Natale et al. [23] found that, among 22 chemical compounds found in the headspace of peach shoots, (*Z*)-3-hexen-1-yl acetate and (*Z*)-3-hexen-1-ol were most attractive to females of *G*. *molesta*, and these two are also found in apple shoots. Rothschild and Vickers [10] found that mated females of *G*. *molesta* were attracted to both peach and apple in dual choice tests, and preferred both peach and apple shoots for egg laying [32]. In our results, first-instar larvae of *G*. *molesta* also preferred peach fruits, although *G*. *dimorpha* larvae chose plum fruits most often. Leskey et al. [40] identified sixteen compounds from unripened plum fruits, and, among them, linalool, 2-hexanone, and 3-hydroxy-2-butanone showed great attractiveness for *Conotrachelus nenuphar* (Herbst) (Coleoptera: Curculionidae). Nevertheless, the chemical compounds in peach and apple are not similar [23], and this may have caused the different choices made by the two species. Jung and Kim [19] found that *G*. *molesta* and *G*. *dimorpha* are genetically fully distinct species, even if morphologically similar.

The duration of the egg stage of *G*. *molesta* was not different among the fruits tested, similarly to Du et al. [8], who couldn’t find any difference in egg duration on either shoots or fruits between peach and pear. But, the duration of the larval stage was significantly shorter when larvae were reared on peach or plum rather than apple, which is similar to Myers et al. [25] and Najar-Rodriguez et al. [14], who found that larvae of *G*. *molesta* develop faster on peach than on apple, on both shoots and mid-stage fruits. Du et al. [8] also found that larval development was shorter on both shoots and fruits of peach than on pear when the fruit diameters were 18.1 ± 0.4 and 62.5 ± 3.4 cm.

The duration of the egg stage of *G*. *dimorpha* was also not different among the fruits, but the larval stage was significantly shorter in larvae reared on plum compared with apple. There are no past studies of development of *G*. *dimorpha* on different hosts, but, similar to *G*. *molesta*, larval development in our study was shorter on plum than apple. Therefore, immature fruits of plum may be the best food source for both *G*. *molesta* and *G*. *dimorpha*. This is also supported by other data where the boring rate of first-instar larvae of *G*. *molesta* was higher on peach or plum than apple, while the boring rate of first instars of *G*. *dimorpha* was higher on plum or apple than peach [32, 34]. Furthermore, the fecundity of both *G*. *molesta* and *G*. *dimorpha* reared on plum showed the highest values. Despite the higher behavioral response to peach and better development on peach of *G*. *molesta*, we found negative effects of peach during reproduction without any clear explanation. Possibly, *G*. *dimorpha* might be more negatively affected by the greater hardness of immature peach, as the boring rate of the first instars is reduced by harder fruit tissues [8]. *Grapholita molesta* might not get proper nutrition from the immature peach fruit with such hard tissue. Thus, as in *G*. *dimorpha*, the boring rate of first instars and the rate of exiting by mature larvae from fruits was lowest on peach, and the only female that did emerge did not lay any eggs.

Life tables are essential tools for determining the critical life stages during arthropods’ development and their influence on the overall population structure [41]. This is the first fertility life table of *G*. *molesta* and *G*. *dimorpha* reared on different immature fruits. The results indicate that immature plum and apple fruit are the most suitable food source for both *G*. *molesta* and *G*. *dimorpha*. Nevertheless those three immature fruits may not be available in the same time in the field condition. More detailed studies on quality of immature fruits, e.g., hardness and sugar content, may be needed to explain the asymmetry in the life table parameters of *G*. *molesta* and *G*. *dimorpha*.

## Supporting information

S1 File Development time and survival rate, fecundity, life table parameters, and choice test. (XLSX)
