# Correction: Development and fecundity performance of *Grapholita molesta* and *Grapholita dimorpha* (Lepidoptera: Tortricidae) on different immature fruits

**DOI:** 10.1371/journal.pone.0219401

**Published:** 2019-07-02

**Authors:** The PLOS ONE Staff

The images for Figs 1, 2, 4 and 5 are incorrectly switched. The image that appears as Fig 1 should be Fig 5, and the image that appears as Fig 5 should be Fig 1. The image that appears as Fig 2 should be Fig 4, and the image that appears as Fig 4 should be Fig 2. The figure captions appear in the correct order. The publisher apologizes for the error.

**Fig 1:**
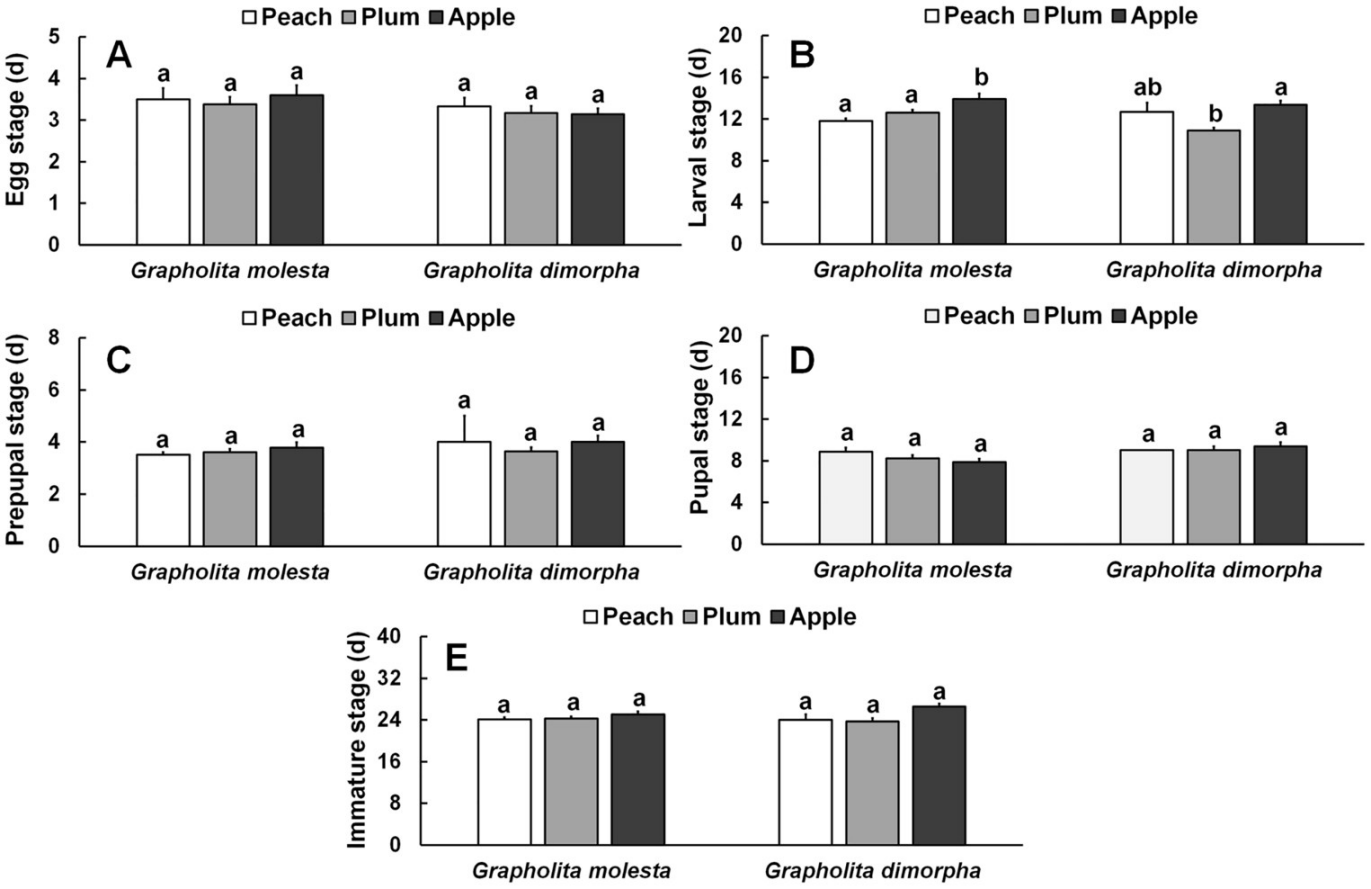
Duration (d ± SE) of each stage of *Grapholita molesta* (n = 76 for peach, n = 52 for plum, and n = 36 for apple) and *Grapholita dimorpha* (n = 30 for peach, n = 32 for plum, and n = 30 for apple) reared on different fruits under laboratory conditions. A) Egg stage, B) Larval stage, C) Prepupal stage, D) Pupal stage, and E) Immature stage.

**Fig 2:**
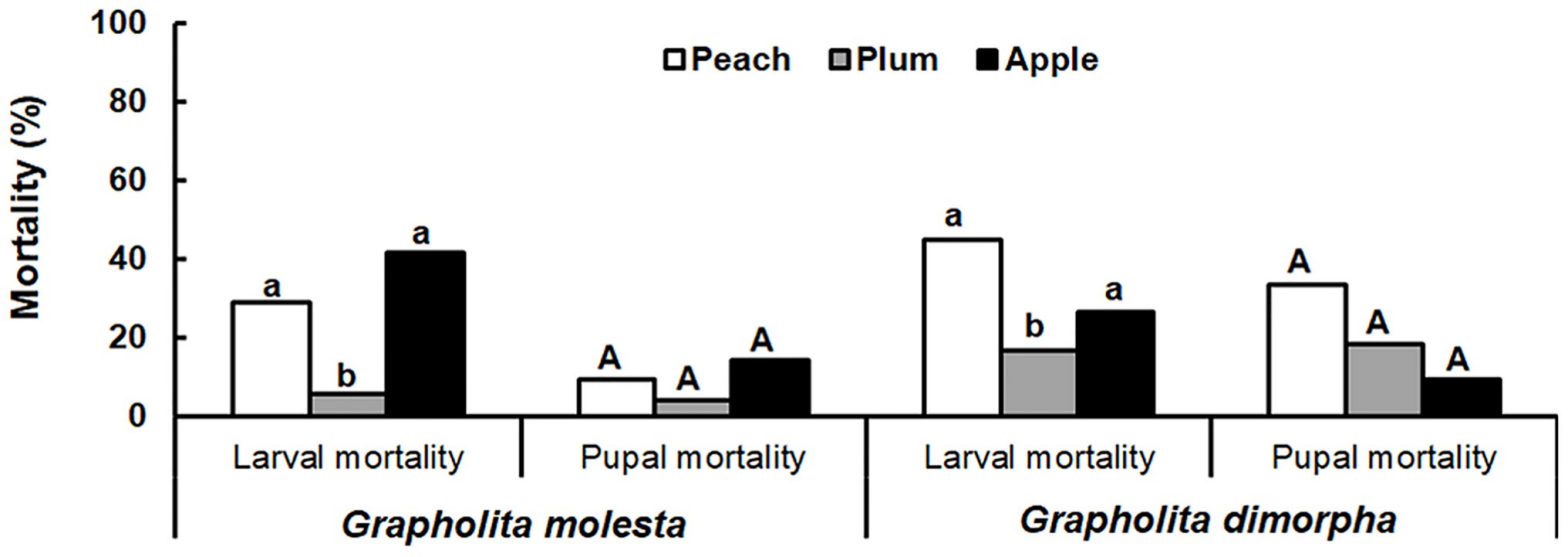
Larval and pupal mortality of *Grapholita molesta* and *Grapholita dimorpha*.

**Fig 4:**
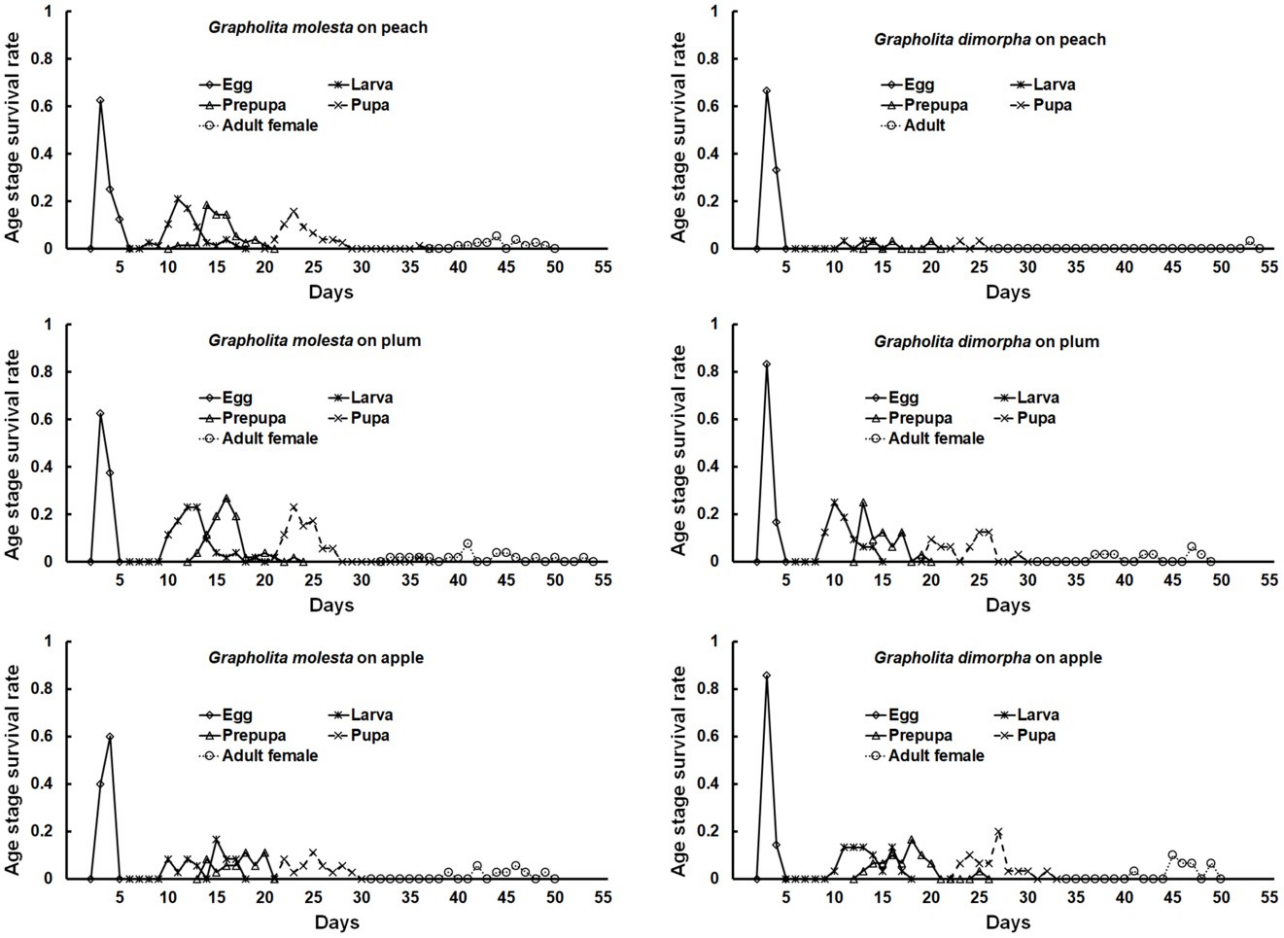
Age-stage survival (*Sxj*) of *Grapholita molesta* and *Grapholita dimorpha* on peach, plum, and apple fruit.

**Fig 5:**
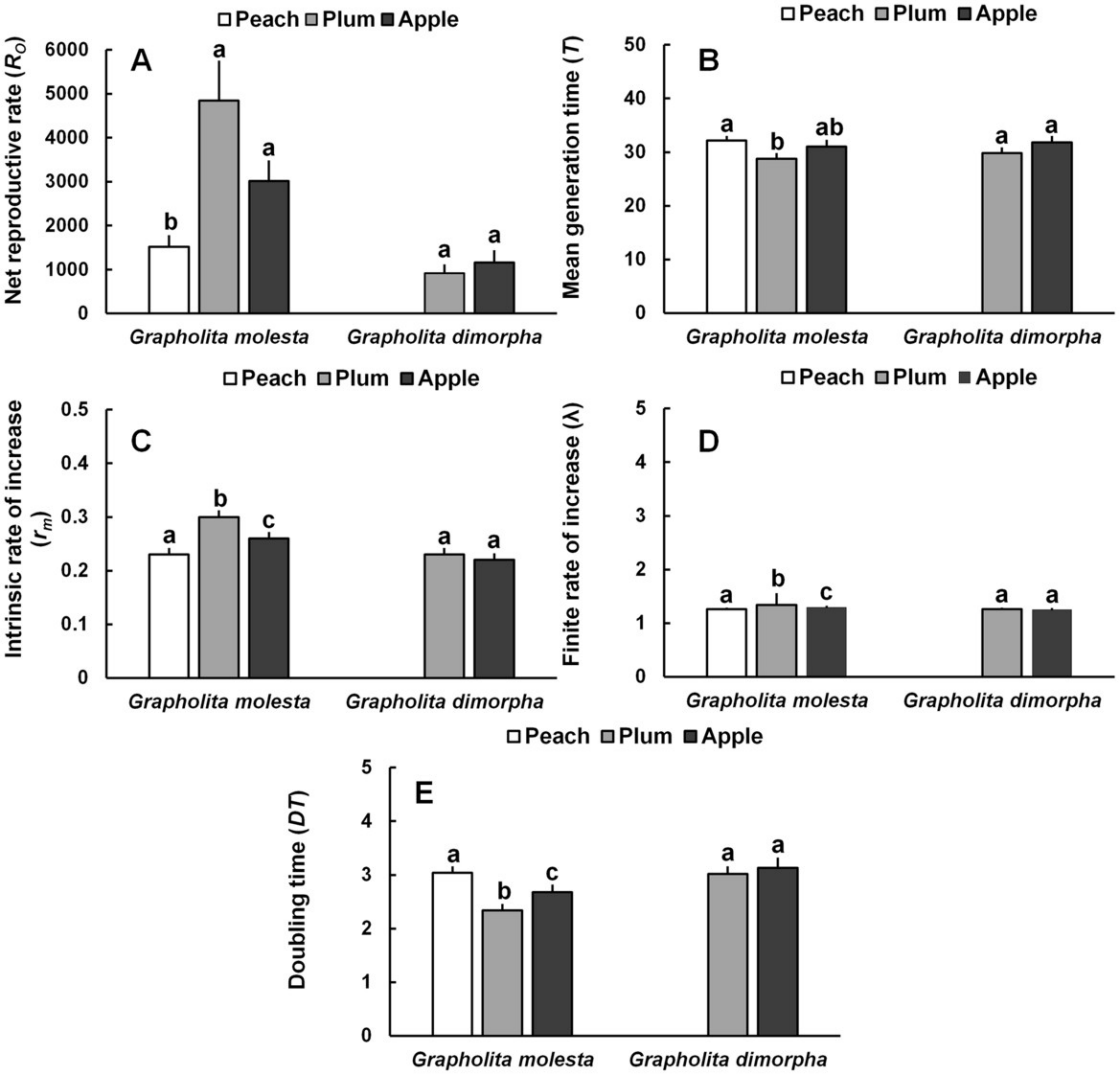
Life table parameters of *Grapholita molesta* and *Grapholita dimorpha* reared on different fruits. A) Net productive rate, B) Mean generation time, C) Intrinsic rate of increase, D) Finite rate of increase, and E) Doubling time. Means followed by the same letter in a column are not significantly different, Student’s *t*-test for pairwise group comparison at P<0.05. *R*_*O*_, Net reproductive rate; T, Mean generation time; DT, Doubling time; λ, Finite rate of increase; *r*_*m*_, Intrinsic rate of increase.
